# Protection against SHIV Challenge by Subcutaneous Administration of the Plant-Derived PGT121 Broadly Neutralizing Antibody in Macaques

**DOI:** 10.1371/journal.pone.0152760

**Published:** 2016-03-31

**Authors:** Yvonne J. Rosenberg, David C. Montefiori, Celia C. LaBranche, Mark G. Lewis, Markus Sack, Jonathan P. Lees, Xiaoming Jiang

**Affiliations:** 1 PlantVax Corporation, Rockville, Maryland, 20850, United States of America; 2 Department of Surgery, Duke University Medical Center, Durham, North Carolina, 27710, United States of America; 3 Bioqual Inc., Rockville, Maryland, 20850, United States of America; 4 Institute of Molecular Biotechnology, RWTH Aachen University, 52056, Aachen, Germany; Centers for Disease Control and Prevention, UNITED STATES

## Abstract

Intravascular delivery of broadly neutralizing antibodies (bnAbs) has shown promise for prevention and treatment of HIV infection. However, multiple IV administrations in geographic locations with poor accessibility to medical care have practical limitations. We have assessed the efficacy of plant-derived PGT121 delivered subcutaneously (SC) against pre-and post-intravaginal challenge using a rigorous SHIV-SF162P3 macaque protection model. SC administered PGT121 exhibited a longer serum half-life than IV administration and was more consistent than intramuscular delivery. A dose of 3.5mg/kg PGT121 prevented infection at a minimum ID50 neutralization titer of 1:295 while 5mg/kg protected five of six macaques when delivered immediately post-challenge. These results suggest the utility of plant-derived bnAbs delivered SC for HIV prevention.

## Introduction

Passive administration of the potent HIV bnAb PGT121 when delivered to macaques intravenously (IV) has been shown to be efficacious in preventing SHIV infection or reducing viral load (VL) [1,2]. Similarly, transient suppression of viremia in HIV-infected humans following a single IV infusion of the bnAb 3BNC117 has been observed [3], demonstrating the safety and proof of concept of this modality in humans. In addition, passively IV administered bnAbs have been shown to synergize with autologous naturally arising anti-HIV antibodies [4] and reduce viral rebound after termination of antiretroviral drug therapy [5]. For the prevention of mother-to-child transmission (MTCT) in resource poor countries, IV injections are not practical and alternate routes of delivery should be considered for optimal benefit and protection. In this context, virtually all human vaccines currently on the market are administered via subcutaneous (SC) or intramuscular (IM) routes with SC delivery being most commonly used when high doses of gamma globulins or biologics/drugs are required. Similarly, SC administration of bnAbs may also be the preferred route of delivery in the case of MTCT or high-dose-immunotherapy (~30mg/kg).

The N332 glycan-dependent PGT121 was chosen here to assess the efficacy of SC delivery of a potential immunotherapeutic HIV candidate because of its breadth and high potency of neutralization, its lack of immunogenicity in macaques, and its very high expression levels reaching 1.6g/kg using a transient *N*.*benthamiana/p19* plant system [6]. This plant platform offers advantages in terms of speed and versatility, human pathogen-free nature and low-tech requirements, and has been used to produce >10 potent in-vivo characterized HIV bnAbs glycoforms with neutralizing activity similar to their mammalian cell counterparts [6,7]. The potential for low production costs, combined with a more compliant SC administration in resource poor settings, offers a potential path to provide PGT121 and other passive HIV immunotherapies to those in need.

## Methods

### Non-human Primates

Rhesus macaques (*Macaca mulatta*) and African Green monkeys (*Chlorocebus pygerythrus*) were housed at BIOQUAL's housing facilities in Rockville, MD. Care and husbandry of all non-human primates were provided in compliance with federal laws and guidelines as well as in accordance with recommendations provided in the NIH guide and other accepted standards of laboratory animal care and use. BIOQUAL is accredited by the Association for the Assessment and Accreditation of Laboratory Animal Care, (AAALAC file #624) and holds an Assurance on file with the National Institute of Health, Office for Protection of Research Risks as required by the US Public Health Service Policy on Humane Care and Use of Laboratory Animals with a PHS Animal Welfare Assurance File Number #A-3086-01. Animals were sedated with ketamine or telazol for all technical procedures. Ketamine was given IM in amounts necessary for short-term procedures such as blood drawing.

Animals were housed in accordance with the recommendations of the Association for Assessment and Accreditation of Laboratory Animal Care International Standards and with the recommendations in the Guide for the Care and Use of Laboratory Animals of the United States—National Institutes of Health. The Institutional Animal Use and Care Committee of BIOQUAL approved these experiments. When immobilization was necessary, the animals were sedated intramuscularly with 10 mg/kg of Ketamine HCl (Parke-Davis, Morris Plains N.J.) before any direct handling or procedures. All efforts were made to minimize suffering. Details of animal welfare and steps taken to ameliorate suffering were in accordance with the recommendations of the Weatherall report, “The use of non-human primates in research”. Animals were housed in an air-conditioned facility with an ambient temperature of 21–25°C, a relative humidity of 40%–60% and a 12 h light/dark cycle. Animals were socially housed when possible or individually housed if no compatible pairing could be found. The animals were housed in suspended, stainless steel, wire-bottomed 6 sq ft cages and provided with a commercial primate diet and fresh fruit and vegetables twice daily with water freely available at all times. Social housing, toys, foraging equipment and mirrors were provided. Animals were monitored at least twice daily for behavior, food intake, activity, and overall health by trained technicians. No macaques were euthanized and all animals were returned to the colony for recycling.

### Antibody production in plants

Monoclonal antibody PGT121 was produced in *N*. *benthamiana* using a *Agrobacterium*-mediated transient gene expression system as described previously [7]. Briefly, codon-optimized heavy and light chain DNA fragments were synthesized and separately cloned into pTRAk plant expression vector. In addition, the silencing suppressor protein p19 from tomato bushy stunt virus was also cloned into a separate pTRAk vector. Six-eight week old plants were co-infiltrated with the recombinant *Agrobacteria* suspension containing the three expression plasmids. After infiltration, plants were incubated at 20°C with 16/8 hour light cycles. At 10–12 days, soluble proteins were extracted and purified by protein A and MEP HyperCel chromatography with a recovery of 1.3 g/kg.

### Non-human primate studies

For the pharmacokinetic studies, two African Green monkeys (~4kg) were injected with 5 mg/kg of plant-derived PGT121 either SC in the back or IM in the thigh, bled from the femoral artery at 0 to 14 days and assessed for levels of PGT121 by both neutralizing antibody activity (ID50) using TZM-bl cells as previously described [8] and ELISA. IC50 neutralization titers are purified antibody concentrations, and ID50 are serum dilutions, at which relative luminescence units (RLU) were reduced by 50% compared to RLU in virus control wells after subtraction of background RLU in cell control wells. ELISA assays were performed using 96-well Immuno Module plates (Nunc) coated with anti-human kappa LC (50μL of 1μg/mL) (SIGMA K3502) or with 1μg/mL of either CHO-derived monomeric HIV BaL-gp120 (NIH HIV Reagent Program) or m.CONgp140 env (a kind gift of Dr Bart Haynes, Duke University, NC) as previously described [6].

Two SC protection studies were conducted using 3-6kg Indian rhesus macaques (*Macaca mulatta*). In the first study, macaques were injected SC with 3.5–7.1mg/kg of PGT121, 24h prior to intravaginal challenge with a high dose (1700 TCID) of SHIV SF162P3 that was expected to infect all control animals after a single challenge. For intravaginal challenge, anesthetized macaques were administered SHIV SF162P3 using a non-leuer-lock syringe inserted ~2 cm into the vaginal vault.

In the second study, the same macaques were injected SC with 5mg/kg of PGT121 at 30–60 mins post-vaginal challenge with SHIV SF162P3 (1700 TCID. The potency of the plant-derived PGT121 against the rhesus (R157) PBMC-derived SF162P3 stock used for challenge was 0.08 ug/ml; similar to the IC50 of CHO-derived PGT121 (0.15 ug/ml). Protection was assessed using a viral RNA assay as described [9]. Fisher’s exact test was performed using the R statistical package [10].

## Results

### Pharmacokinetics of plant-derived PGT121 following subcutaneous and intramuscular administration

**Fig 1:**
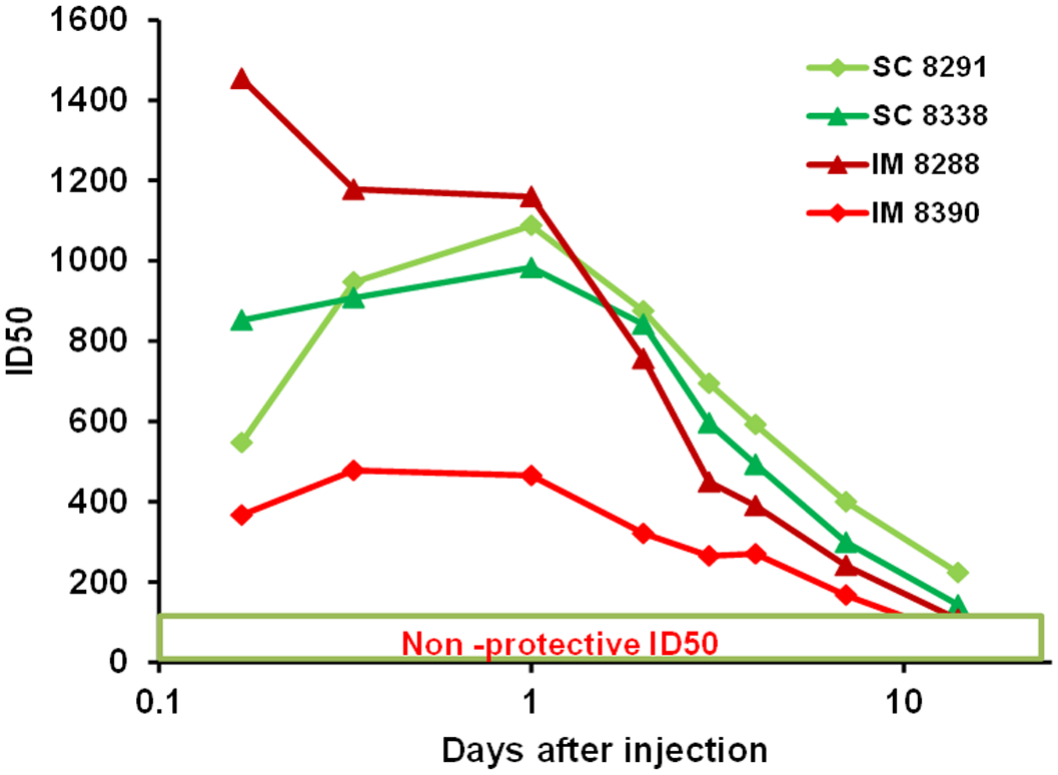
Circulatory clearance profiles of 5mg/kg of plant-derived PGT121 in African Green monkeys following SC and IM injections. Neutralizing ID50s were assessed at different times after injection using a pseudovirus-based TZM-bl assay against the Tier 2 SHIV-SF162P3.

In order to establish the time of peak PGT121 levels (Tmax) following parenteral administration and thus the time of challenge to assess optimal protection, preliminary studies were performed in which two African Green monkeys each received 5mg/kg of PGT121 by either SC or IM delivery and the pharmacokinetics monitored from 4 hr to 14 days. Fig 1 compares the serum neutralizing ID50 clearance profiles for each monkey using a pseudovirus-based TZM-bl assay against SHIV SF162P3. Cmax data were also assessed from the same samples by ELISA. Results indicate that each of the monkeys receiving SC injections (#8338, #8291) exhibited similar pharmacokinetics with Cmax of 60μg/ml and 80μg/ml at Tmax of ~24-30hr. By contrast, clearance profiles and Tmax in monkeys injected IM differed widely. Thus, monkey #8288 exhibited a T max of 4hr and a Cmax of 110ug/kg while the corresponding values for #8390 were 16μg/ml at a Tmax ~18-24hr. Based on minimum predicted protective neutralizing titers, these data suggest that SC injection of PGT121 (5mg/kg) will be protective within a range of <4hr to >14 days.

### Efficacy of protection of PGT121 delivered by the subcutaneous route

**Fig 2:**
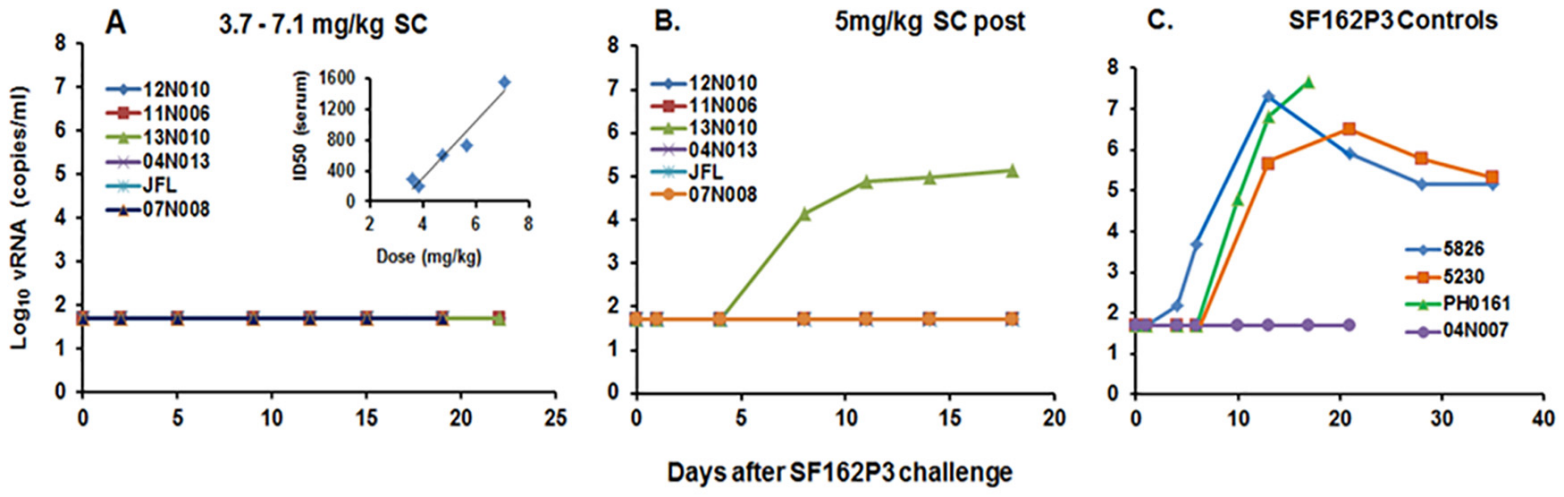
Protection by SC-administered plant-derived PGT121 against intravaginal SHIVSF162P3 challenge in rhesus macaques as measured by log 10 vRNA (copies/ml of plasma). (A) Protection in 6/6 macaques that received 3.7–7.1 mg/kg doses of PGT121 given 24 hr prior to SHIV SF162P3 challenge. Insert shows a linear correlation between ID50 and dose (3.5–7.1 mg/kg) at 24 hr; ID50 values at doses of 3.86, 4.7 and 5.6 mg/kg were 1:202, 1: 612 and 1: 738 respectively). (B) Protection in 5/6 macaques administered 5 mg/kg SC immediately after SF162P3 challenge. (C) Four control macaques received no passive bnAb. Application of Fisher’s Exact Test showed that protection of animals given PGT121 prior to challenge (panel A) compared to the control group (panel C) was statistically significant (p = 0.03), while protection of animals given PGT121 after challenge was not (p = 0.19).

Based on the ID50 clearance profiles, the efficacy of PGT121 given SC to protect against SHIV SF162P3 challenge at the Cmax was assessed in an initial dose-finding protection study. Thus, doses of 3.5–7.1mg/kg 24hr prior to intra-vaginal challenge with SF162P3 (1700 TCID) resulted in sterilizing protection in all six rhesus macaques (Fig 2A). This protection reached statistical significance compared to the control group by the Fisher’s Exact Test (p = 0.03). This is consistent with the serum PGT121 neutralizing levels at all doses against SHIV-SF162P3, measured just prior to challenge (inset in Fig 2A) with the lowest dose of 3.5mg/kg resulting in an ID50 of 1:295 (12N010) and reaching an ID50 of 1:1,565 at the 7.1mg/kg dose.

In order to mimic aspects of MTCT wherein infants born to HIV+ mothers may be injected immediately after birth, two months later these same six protected animals were administered 5 mg/kg of PGT121 SC immediately (30–60 mins) after intravaginal challenge with 1700 TCID SF162P3; resulting in protection in 5 of the 6 macaques at day 18 (Fig 2B). As observed previously in several published studies [11–13], one of the four untreated SHIVSF162P3-infected controls was not infected even at a high TCID (Fig 2C). Protection of animals given PGT121 after challenge was not significantly different from the control group (p = 0.19, Fisher’s Exact Test), likely due to the one control animal that was not infected during challenge with SHIV-SF162P3.

## Discussion

Efficacious prophylactic HIV immunotherapy may require multiple administrations and will require the retention of potent bnAbs at therapeutic levels in the circulation for prolonged periods to counteract high early viral replication without rapidly inducing either anti-idiotypic immune responses [6,14] or viral escape mutants. In terms of pharmacokinetic parameters following different routes of delivery shown in Fig 1, the very high potency of recent HIV bnAbs means that their Cmax values, highest after IV infusion, are less critical than their half-life values and that the larger MRT and T1/2 after parenteral injections, administered as a single injection or as small repeated injections, may afford a more practical delivery system than IV delivery, especially if bnAbs can be self-administered.

Direct comparison of the pharmacokinetics following SC and IM delivery indicated that the Cmax and Tmax were consistent in both monkeys after SC administration with longer retention at the same dose than IV injection shown previously [6]. By contrast, Cmax and Tmax varied considerably following IM injections; with monkey #8288 exhibiting values more closely resembling an IV injection [6] with a Cmax of 115ug/ml (not shown). Although these preliminary studies used small numbers of macaques, the consistency of Tmax following SC delivery and the inconsistent absorption following IM injections has been observed previously in 8 macaques following injection of high doses of another large therapeutic protein [15]. Whether the more rapid exit from the injection site into the blood reflects a more vascularized muscle or whether some IM injections have the potential to damage tissue and blood vessels and promote faster draining is not clear.

Based on the more consistent clearance profiles, protection studies against SHIV SF162P3 challenge were performed using SC delivery of PGT121. Since SC delivery of PGT121 has not been reported and Cmax values following SC injections are known to be lower than those following IV injections, a dose of ~3mg/kg was initially used. Fig 2 indicates that the six macaques that received PGT121 doses of 3.5–7.1mg/kg SC exhibited a good dose-response curve at 24hr when measured as neutralizing ID50s against this SHIV isolate, demonstrating near complete absorption from the injection site. In addition, ID50s against Tier 2 RHPA4259.7 (PGT121 IC50:0.03μg/ml) and Tier 1 SHIV-BalP4 (IC50:0.07μg/ml) also correlated well with dose in these macaques (not shown). All six macaques remained negative following challenge with SHIV SF162P3, confirming that a circulating ID50 titer of <1:292 (observed at the lowest 3.5mg/kg dose used) is protective against intravaginal challenge and suggests that at lower SC doses the protective neutralizing level will be similar to the calculated minimum protective neutralization titer in plasma of 1:100 required to prevent viral acquisition following intra-rectal challenge [16]. Although SC injections do not reach peak Cmax until 24-30hr and a small window of vulnerability may exist early (1-2hr?), 5/6 macaques were protected when PGT121 was administered SC within 30mins after challenge. The inability of one untreated control macaque to become infected has been noted previously where SHIV-SF162P3 challenge either failed to infect or was highly controlled [11–13]. It should be noted that three of the five protected macaques produced no anti-PGT121 responses following two injections while two macaques exhibited atypical short-lived primary and secondary ant-PGT121 responses similar to those previously reported [6]; possibly reflecting polyreactivity or environmental stimulation.

Unlike IV injections, SC and IM administration of macromolecules (MW >2,000) and micro-particles (100 nm in diameter) favor drainage into the more open capillaries of lymph from the interstitial spaces via cleft-like openings; lymphatic transport being the primary means with molecules of MW >16,000 in sheep studies [17–19]. For this reason, the T1/2 is longer following SC delivery than IV delivery. This prolonged absorption, reduces Cmax and delays peak Tmax values, which in the case of HIV bnAbs may lead to longer periods of protection or VL reduction. In these studies, SC delivery of PGT121 has been shown to be an efficacious route of delivery in terms of protecting both pre-and post- SHIV challenge and may be further enhanced using targeted Fc mutations [20] affording a more practical delivery system than IV.
